# Five-year Follow-up of Chronic Hepatitis B Patients Immunized by Nasal Route with the Therapeutic Vaccine HeberNasvac

**DOI:** 10.5005/jp-journals-10018-1279

**Published:** 2019-02-01

**Authors:** Guillermo Fernández, Ana L Sanchez, Everardo Jerez, Luis E Anillo, Freya Freyre, Jorge A Aguiar, Yamila Leon, Zurina Cinza, Pablo A Diaz, Nelvis Figueroa, Verena Muzio, Gerardo G Nieto, Yadira Lobaina, Arístides Aguilar, Eduardo Penton, Julio C Aguilar

**Affiliations:** 1 Department of Gastroenterology, “Abel Santamaría” Provincial Hospital, Pinar del Rio, Cuba; 2 Department of Vaccine, Biomedical Research, Center for Genetic Engineering and Biotechnology, Havana, Cuba

**Keywords:** Chronic hepatitis B, HeberNasvac, Therapeutic vaccine.

## Abstract

A novel therapeutic vaccine for chronic hepatitis B (CHB) treatment comprising the recombinant hepatitis B surface (HBsAg) and nucleocapsid (HBcAg) antigens has been developed. Preclinical and clinical trials (CT) evidenced safety and immunogenicity in animal models as well as in phases I, II, and III clinical trials.

A phase I CT has conducted in Cuba in 6 CHB patients refractory or incomplete responders to α-IFN. Patients were immunized ten times every two weeks *via.* nasal spray, with 100 ug HBsAg and 100 ug HBcAg. Clinical efficacy was monitored by assessing the levels of hepatitis B virus deoxyribonucleic acid (HBV DNA), alanine aminotransferase (ALT), HBeAg, and anti-HBeAg seroconversion as well as by qualitative/ quantitative HBsAg serology during this period.

After a 5 year follow-up,HBeAg loss was verified in the three HBeAg (+) patients, in two cases with seroconversion to anti-HBeAg. A reduction to undetectable viral load was observed in 5 out of 6 patients, and in two cases HBsAg seroconversion was also detected. ALT increases above the 2X upper limit of normal (ULN) were only detected in HBeAg (+) patients and associated with HBe antigen loss. All patients had stiffness levels below 7.8 KPa by Fibroscan assessment at the end of this period.

Although only a few patients were enrolled in this study, it seems that HeberNasvac may maintain some of the therapeutic effects for a prolonged period.

**How to cite this article:** Fernandez G, Sanchez AL, Jerez E, Anillo LE, Freyre F, Aguiar JA, Leon Y, Cinza Z, Diaz PA, Figueroa N, Muzio V, Nieto GG, Lobaina Y, Aguilar A, Penton E, Aguilar JC. Five-year Follow-up of Chronic Hepatitis B Patients Immunized by Nasal Route with the Therapeutic Vaccine HeberNasvac. Euroasian J Hepatogastroenterol, 2018;8(2):133-139.

## INTRODUCTION

With an estimated of more than 240 million people infected worldwide, CHB remains a major public health problem. The CHB is considered a major factor for serious liver diseases such as cirrhosis, hepatocellular carcinoma, and related complications.^1^ The current antiviral therapies have limited off-treatment efficacy, requiring long-term continuous and expensive treatments and follow-ups; however, definitive viral elimination is infrequent).^2^

Immunotherapeutic strategies target the induction of CD4 and CD8 T-cell responses and the stimulation of pro-inflammatory cytokines capable of controlling viral replication. Several vaccine candidates have been clinically tested in CHB patients, although their efficacies have been limited or inconclusive.^3^

A novel vaccine formulation called HeberNasvac has been tested in preclinical and clinical studies. This product was originally developed as a nasal vaccine for CHB immunotherapy that comprises the hepatitis B virus (HBV) surface and core recombinant virus-like particles (VLP).^4,5^ Preclinical studies including immuno-toxico-logical evaluations using HBsAg-transgenic mice have been completed.^6,7^ HeberNasvac was very immunogenic in mice after intranasal (IN) or parenteral administrations inducing high antibody titers, potent proliferative responses and the secretion of gamma interferon by spleen cells.^4-7^ The formulation also demonstrated its safety and preliminary immunogenicity in a phase I CT in healthy volunteers.^8^ Phase I, II and III CT^9,10^ conducted in Bangladesh, a country with intermediate to high prevalence of CHB^11,12^ demonstrated the safety and efficacy of this vaccine in treatment-naïve CHB patients after IN and SC administration.

Although major studies with HeberNasvac were conducted in treatment-naïve CHB patients immunized by both IN and SC routes, further studies in different patient groups pharmacological studies have shown that the nasal route deserves further attention^13^ The present work describes the retrospective analysis of the virological, biochemical and serological variables of CHB patients during 5 years after only IN immunization with HeberNasvac.

## MATERIALS AND METHODS

### Patients

The CHB patients enrolled in the follow-up study were those included in the phase I CT aimed at studying the safety and preliminary efficacy of HeberNasvac. Patien-tsin the age range of 18 to 65 years old, non-responders, refractory or incomplete responder to α-IFN treatment, with HBsAg-positive serology and ALT values higher than the upper limit of normality (ULN) in the previous 6 months, were recruited for the phase I CT. Patients were excluded from enrollment if they had acute or chronic respiratory diseases, HCV or HIV infection markers. A woman in fertile age using a hormonal contraceptive, pregnant or lactating were also excluded. Other causes of exclusion are non-compensated chronic diseases and levels of ALT or AST higher than 500 U/L in any time during the study.

### Therapeutic Immunization

HeberNasvac formulation administered during the phase I CT comprised a mixture of 100 μg Pichia pastoris derived recombinant HBsAg subtype adw2 (CIGB, Cuba) and 100 μg *Escherichia coli* purified recombinant full length (183 amino acids) HBcAg variant (CIGB, Cuba). HBsAg was produced as a 22 nm particle to more than 95% purity at the CIGB production facilities as a component of the commercial anti-HBV vaccine, heberbiovac-HB. The HBsAg only comprises the S protein, expressed and purified as a non-glycosylated form.^14^ HBcAg was purified from *E. coli* strain W3110, transformed with a plasmid containing the entire core antigen gene under the control of the tryptophan promoter.^15^ The resulting HBcAg had a purity superior to 95% and a mean size of 28 nm as characterized by electron microscopy (EM) analysis.^16^ The antigens were formulated in a phosphate-saline buffer, no adjuvant or preservative was used, rendering a sterile, aqueous and transparent liquid. Each vial contained 1.6 mL of the vaccine formulation. All study products were stored in 6R vials at 2 to 8° C until use. Patients received ten doses, every 14 days. A volume of 1mL of the vaccine formulation was administered by IN route using a VP7D pump (Valois, France). The total volume was split in eight actuation of 125 μL, alternatively applied by each nostril. A five minutes pause between actions in the same nostril was implemented. The vaccine was administered to the patient seated with the head tilt back.

### Safety-related Evaluations

Adverse events (AE) were actively recorded during phase I study period demonstrating that HeberNasvac was safe and well tolerated (reviewed in 17). AE was passively recorded during the following 5 years. Different parameters of basic hematology and blood chemistry were periodically evaluated during and after immunizations as well as during follow-up to explore the effect of the immune response in the short and long term. Liver Stiffness was used to assess fibrosis, and it was conducted at the Institute of Gastroenterology, Havana, using a FibroScan 502 model (Echosens, France).

### Efficacy Related Evaluations

Efficacy variables were monitored during and after immunizations by the analysis of the viral load, ALT, liver function tests (LFT), qualitative serological tests (HBsAg and HBeAg and their corresponding antibodies) and quantitative HBsAg serology. Follow-up evaluations were conducted every 6 months after the end of treatment (EOT) until five years.

The viral load determinations were carried out using the HBV in-house PCR system developed and validated at the Center for Genetic Engineering and Biotechnology and Institute of Tropical Medicine “Pedro Kouri,” Havana.^17,18^ This system has a detection limit of 20 copies/ mL.

The ALT levels were assessed during the five years study period. The value of 49 U/L was the ULN according to the system commercially available at the “Abel Santamaria” Hospital, Pinar del Rio Province, Cuba. Local treatment guidelines recommend that patients should be treated after two consecutive evaluations with more than 2X ULN in three months.

The serological response was assessed by the loss of HBeAg/HBsAg, or HBeAg/HBsAg seroconversion, defined by the loss of each antigen and appearance of the specific antibodies at the same time. The qualitative HBsAg, anti-HCV and anti-HIV determinations were done using SUMA commercial kits (TecnoSuma, Cuba). The HBeAg and anti-HBe markers were measured by an ELISA commercial diagnostic kit (Diasorin, Italy), and the quantitative HBsAg and anti-HBsAg were conducted using CIGB in-house validated systems.

### Data Analysis and Statistical Considerations

All examinations and graphs were performed using Prism 5.0 statistical software application. The objective of the present study was exploratory. The retrospective analysis of six patients was conducted only to estimate the individual behavior of each patient on time and preliminarily assess the effect of the nasal vaccine on disease progression.

## RESULTS

### Patients

A total of 6 CHB patients were enrolled in the five years follow-up. These patients were treated with α-IFN (Heberon Alfa, Heber Biotec, Cuba), more than 10 years before HeberNasvac treatment, and were recruitedinto the study after obtainingwritten informed consent. Table 1 summarizes the patient’s baseline characteristics.

Few relevant characteristics of this group of patients are: patient 1 was previously (12 years ago) treated for leukemia, patient 6 started the immunizations with more than 60 years old, and patient five declared alcohol intake. In general, the six patients had a history of 12 or more years with the disease and intermittent ALT increases before HeberNasvac administration, evidencing an incomplete response to α-IFN therapy.

### Safety and Clinical Adverse Events

All recruited subjects completed the immunization schedule, except one who missed the last administration due to treatment non-related causes. The vaccine was clinically safe during immunization, and there was no severe event reported by any of the patients during the follow-up period. No cases of hepatic, renal or bone marrow dysfunctions during the immunotherapy or after the treatment. No hospitalization was required. No pathologic deviations in hematological variables or any other parameters attributable to vaccination were recorded in the follow-up period. In general, during the five years post-treatment, the recorded AE were mild and disappeared without medical intervention, as well as during the vaccination period. Table 2 includes the summary of the AE per dose.

**Table 1:** **Table 1:** Patient demographics at the start of immunizations

				*HeberNasvac^®^*			
*Variables*				*N*		*%*	
N				6		100.0	
Sex		Female		3		50.0	
		Male		3		50.0	
		White		3		50.0	
Ethnicity		Black		2		33.3	
		Mixed		1		16.7	
		N		6			
Age		Mean ± SD		43.3 ± 9.9			
		Median ± RQ		40 ± 10			
		N		6			
Weight (Kg)		Mean ± SD		67.5 ± 23.1			
		Median ± RQ		63.2 ± 37.3			
		(Minimum; Maximum)		(44; 108)			
		N		6			
BMI (Kg/m^2^)		Mean ± SD		22.9 ± 4.9			
		Median ± RQ		22.8 ± 8.9			
		(Minimum; Maximum)		(17.8; 30.9)			

### Evaluation of the Major Efficacy Variables

**Fig. 1:**
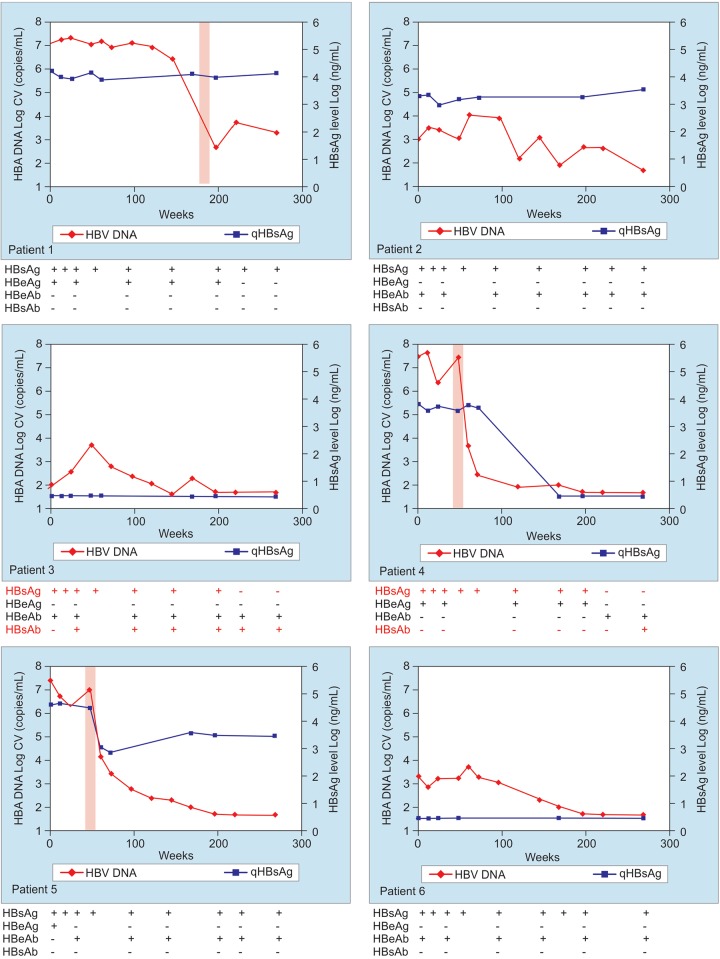
HBV DNA and quantitative HBsAg on time during the administration and following a period of 5 years follow-up. The results of the serological determinations are presented below their corresponding graph for each patient (Patient 1 to 6). The timeframe corresponding to the ALT flare is represented by a shadow area

All patients finished the follow-up period with undetect-able (5 out of 6) or low viral load (1/6). The viral load was sustainedly undetectable in four patients during at least one year (3 consecutive determinations every 6 months). Low levels of HBV DNA (below 10^4^ copies/mL) were detected in two patients (patients 1 and 2, Fig. 1). Patient 2 sustained viral replication below 10^3^ copies/mL for more than one and half year.

The three HBeAg positive patients eliminated HBeAg, in one case at the end of the immunization in two other cases during the follow-up assessments. In two cases there was seroconversion to anti-HBeAg during the five year follow-up period (Fig 1). The patient that did not seroconvert to anti-HBeAg was the one with long-lasting viremia (above 10^3^ HBV DNA copies/mL) and the history of leukemia (patient 1, Fig 1).

HBsAg loss was detected in two out of six patients as a late serological development. Both patients were anti-HBsAg positive in addition to HBsAg loss. Of these, the patient with detectable anti-HBsAg and concomitant HBsAg serology since week 24, was the one with the lowest HBV DNA level at the start of immunizations and with the best physical conditions (patient 3, Fig. 1).

ALT normalization was present in all HBeAg-negative patients during the study follow-up. In HBeAg-positive patients, isolated flareswere detected followed by strong HBV DNA reductions. The elimination or reduction of HBeAg from blood was temporarily associated to ALT-flares (Fig. 1, patients 1, 4 and 5), although in patient 5 HBeAg seroconversion was detected previous to the flare (week 24).

Fibroscan evaluation of all six patients evidenced the absence of cirrhosis after 5-year follow-up; all patients remained in levels of liver stiffness equivalent to mild fibrosis (below 7.8 KPa). No clinical, biochemical or ultrasound manifestations of cirrhosis were detected at the end of follow-up.

**Table 2:** **Table 2:** Frequency of each adverse event, per dose.

						*Dose*																					
*Variables N=6*						*1st*		*2nd*		*3rd*		*4th*		*5th*		*6th*		*7th*		*8th*		*9th*		*10th*		*Total*	
With at least 1 AE				Yes		5(83.3)		5(83.3)		6(100.0)		4(66.7)		6(100.0)		6(100.0)		6(100.0)		5(83.3)		4(66.7)		5(83.3)		6(100.0)	
				No		1(16.7)		1(16.7)		-		2(33.3)		-		-		-		1(16.7)		2(33.3)		1(16.7)		-	
		Asthenia				3(50.0)		5(83.3)		1(16.7)		1(16.7)		-		1(16.7)		2(33.3)		1(16.7)		1(16.7)		1(16.7)		5(83.3)	
		Headaches				3(50.0)		4(66.7)		1(16.7)		1(16.7)		3(50.0)		3(50.0)		4(66.7)		1(16.7)		-		-		6(100.0)	
		Diarrhea				1(16.7)		3(50.0)		1(16.7)		-		1(16.7)		-		1(16.7)		-		-		-		4(66.7)	
		Light fever				2(33.3)		-		-		1(16.7)		-		-		-		-		-		1(16.7)		3(50.0)	
		Fever				-		-		-		-		1(16.7)		-		-		-		-		-		1(16.7)	
Systemic		Hypothermia				-		-		-		1(16.7)		-		-		-		-		-		-		1(16.7)	
		Malaise				2(33.3)		2(33.3)		1(16.7)		1(16.7)		2(33.3)		2(33.3)		2(33.3)		1(16.7)		2(33.3)		1(16.7)		4(66.7)	
		Nausea				1(16.7)		1(16.7)		-		-		1(16.7)		-		1(16.7)		-		-		-		2(33.3)	
		Local pain				1(16.7)		-		1(16.7)		-		-		-		1(16.7)		-		1(16.7)		1(16.7)		4(66.7)	
		Epistaxis				-		1(16.7)		-		-		-		-		-		-		-		-		1(16.7)	
		Sneezing				3(50.0)		2(33.3)		3(50.0)		4(66.7)		4(66.7)		4(66.7)		4(66.7)		4(66.7)		3(50.0)		3(50.0)		6(100.0)	
		Nasal obstruction				-		-		-		2(33.3)		-		-		-		-		-		-		2(33.3)	
		Odynophagia				1(16.7)		-		-		-		-		-		-		-		-		-		1(16.7)	
Local		Nasal pruritus				2(33.3)		-		1(16.7)		1(16.7)		-		1(16.7)		1(16.7)		-		1(16.7)		1(16.7)		4(66.7)	
		Nasal secretion				3(50.0)		1(16.7)		1(16.7)		1(16.7)		-		1(16.7)		1(16.7)		1(16.7)		2(33.3)		1(16.7)		6(100.0)	

## DISCUSSION

After treatment with HeberNasvac, all patients evidenced a constant improvement in the virological and serological variables associated with CHB. No reversion in HBe/HBs loss or seroconversion was detected during the 5-year follow-up. The performance of the serologi-cal and biochemical variables was consistent with viral load reductions. Taken together, based on major current guidelines,^19-21^ none of the patients required additional therapy after treatment with HeberNasvac.

The HBeAg-positive patients displayed isolated and transient ALT increases; these flares were followed by strong viral reductions, temporarily associated with HBeAg reduction or loss. Such associations were also described in a larger HeberNasvac phase III CT following five IN immunizations.^10^ ALT flares have been well described during the immune activation related to disease resolution after treatment,^22^ or spontaneously during the course of the disease.^23^

The patients of the present study, with a documented history of CHB during more than 12 years, incomplete response to α-IFN therapy one decade ago, and several additional complications (comorbidities, alcohol intake and advanced age), exhibited mild fibrosis at the end of follow-up. This slow progression suggests a liver protective role of the therapeutic vaccine, as previously proposed).^9^ Although the number of patients is limited, the results are consistent with those found in the phase III CT, where the proportion of patients with pre-cirrhosis/ cirrhosis was lower after HeberNasvac vaccination compared to PegIFN treatment.^10^

The present results are also in agreement with pre-clinical pharmacological studies in AAV-HBV transfected mice, demonstrating a high proportion of HBV-specific and multifunctional CD4+ T cells in the liver after repeated IN administration of HeberNasvac.^13^ Phase I/ II study conducted in treatment naïve patients also evidenced that patients PBMC preferentially induced Th1 cytokines after IN immunization.^9^

The specific scenario of patients without disease resolution after conventional treatments, more advanced age or difficult to treat settings should be tested in larger studies. This may be further explored in phase IV clinical trials after HeberNasvac registration or studied in cohort investigational trials. The absence of moderate, serious or severe adverse events, and the evidence of slow progression of fibrosis, further supports the safety of HeberNasvac , complementing previous data from in phase I, I/II and III CT.^8-10^ ALT increases have shown a benign nature as also found in Phase III clinical trial,^10^ with the same pattern of host-derived flares.^24^ These flares have also been considered as “benign flares” in a recently published review and metanalysis.^25^

Present results are relevant taking into account the epidemiology of CHB infection, with a higher prevalence in less developed countries^1^. Poverty is linked to the unsafe use of syringes; even countries with emergent economies are unable to completely control this problem.^26^ Therapeutic vaccination strategies would likely require administering multiple doses to overcome tolerance.^9,10,27-29^ The IN route may be an attractive alternative to control the accidental spread of blood-borne diseases. Specifically, in the field of CHB, the only immu-nomodulatory treatment currently approved worldwide need 48 administrations via SC route.^19-21^ In addition, IN administration may offer qualitative advantages for active and specific therapeutic vaccinations.^13^

In conclusion, HeberNasvac administration by the IN route was not associated with any safety concern 5 years after the end of treatment. Virological, biochemical and serological variables evidenced the efficacy of this product in a difficult to treat a group of patients as well as the absence of disease progression. No reversion was verified during the follow-up period in the main variables under study. Further treatment was not required in any of the patients. Nasal therapeutic immunization of CHB patients deserves further attention. A transition is now progressing from preventive vaccine to a therapeutic implication;^30^ however, the impact of protective immunity versus pathogenic immunity must be analyzed in more details.^31,32^
